# Evaluation of the Genotoxicity and Cytotoxicity of Bioceramic Endodontic Sealers in HepG2 and V79 Cell Lines: An In Vitro Study Using the Comet and Micronucleus Assays

**DOI:** 10.3390/jfb16050169

**Published:** 2025-05-09

**Authors:** Antonija Tadin, Marija Badrov, Danijela Juric Kacunic, Nada Galic, Matea Macan, Ivan Kovacic, Davor Zeljezic

**Affiliations:** 1 Department of Restorative Dental Medicine and Endodontics, Study of Dental Medicine, University of Split School of Medicine, 21000 Split, Croatia; marija.badrov@mefst.hr; 2 Department of Maxillofacial Surgery, Clinical Hospital Centre Split, 21000 Split, Croatia; ivan.kovacic@mefst.hr; 3 Private Dental Clinic, 76571 Gaggenau, Germany; migavac78@yahoo.de (D.J.K.); matea.macan@gmail.com (M.M.); 4 Department of Endodontics and Restorative Dental Medicine, School of Dental Medicine, University of Zagreb, 10000 Zagreb, Croatia; ngalic@sfzg.hr; 5 Department of Prosthodontics, Study of Dental Medicine, University of Split School of Medicine, 21000 Split, Croatia; 6 Division of Toxicology, Institute for Medical Research and Occupational Health, 10000 Zagreb, Croatia; dzeljezi@imi.hr

**Keywords:** bioceramics, biocompatibility, calcium silicate sealers, comet assay, cytotoxicity, endodontics, genotoxicity, micronucleus assay, sealers

## Abstract

Background: The primary objective of this study was to evaluate the cytotoxic and genotoxic effects of calcium silicate-based sealers (BioRoot RCS and MTA Fillapex) compared to an epoxy-based sealer (AH Plus). Materials and methods: The study was conducted in vitro with the cell lines HepG2 and V79 to evaluate cytotoxicity and genotoxicity using the comet and micronucleus assays. Eluates of the materials were tested at two different concentrations (3 cm^2^/mL and 0.5 cm^2^/mL) after an exposure time of 72 h. Data were analyzed using the Mann–Whitney and Kruskal–Wallis tests (*p* < 0.05). Results: At lower concentrations in both cell lines, MTA Fillapex showed no significant difference in the measured comet assay parameters compared to the negative control (*p* > 0.05). In addition, it showed significantly lower genotoxic effects compared to AH Plus for all comet assay parameters, concentrations, and cell lines (*p* ≤ 0.001). BioRoot RCS showed lower primary DNA damage (*p* ≤ 0.001) than AH Plus, only at higher concentrations and in the HepG2 cell line. Concerning the two tested bioceramic sealers, BioRoot RCS showed higher tail intensity values compared to MTA Fillapex (*p* < 0.05). In contrast to the results of the comet assay, BioRoot RCS significantly reduced the number of nuclear buds and nucleoplasmic bridges in the HepG2 cell line compared to MTA Fillapex, whereas reduction in the V79 cell line was only observed for nuclear buds (*p* < 0.05). Both materials increased the number of apoptotic cells compared to the negative control (*p* < 0.05). In comparison to AH Plus, BioRoot RCS and MTA Fillapex significantly reduced the number of cells with micronuclei and increased the number of cells with undamaged chromatin (*p* < 0.05). Conclusions: The findings suggest that MTA Fillapex and BioRoot RCS exhibit superior biocompatibility over AH Plus, as evidenced by their lower cytotoxic and genotoxic effects in vitro. These results support the use of calcium silicate-based sealers in clinical practice, highlighting the need for further studies to evaluate their performance in vivo and their implications for patient safety.

## 1. Introduction

Root canal obturation is an important phase in endodontic therapy, and while the properties of the filling material may contribute to treatment success, clinical outcomes are multifactorial, and are also influenced by factors such as proper diagnosis, effective canal disinfection, adequate shaping, coronal restoration quality, and patient-related variables. An ideal material must provide an airtight seal, ensuring both coronal and apical sealing to prevent microbial infiltration. Furthermore, it should adhere well to dentin and the canal walls, forming a strong bond once set. The material should be dimensionally stable and exhibit antibacterial properties to limit microbial growth. Insolubility in tissue fluids is essential for long-term durability, and biocompatibility ensures safety within the biological environment. Ideally, the material should be bioactive, promoting hydroxyapatite formation for enhanced tissue healing and regeneration [1]. Various root canal sealers are commercially available, differing in their chemical compositions and setting mechanisms. These sealers can be categorized into five primary groups: zinc oxide eugenol (ZOE), calcium hydroxide, glass ionomer, epoxy resin, and bioceramic sealers [2].

The toxicity of root canal sealers is linked to their chemical constituents, such as eugenol, bisphenol A, resin monomers, and by-products like formaldehyde and calcium hydroxide, which may leach over time due to solubility. Though intended to remain in the canal, sealers can extrude into periapical tissues through the apical foramen or lateral canals [3]. When they come into contact with tissue fluids, sealers may dissolve, releasing harmful components that can cause cytotoxic and genotoxic effects to periapical cells. These substances may also enter the bloodstream, causing irritation and inflammation and hindering healing after endodontic treatment [4,5]. The setting time and solubility of sealers are crucial for sealing performance. Extended setting times improve canal penetration, but increase the risk of releasing toxic components, with slow-setting sealers, such as epoxy resins like AH 26 and AH Plus, posing a higher risk [1,6]. Bioceramic sealers, like MTA Fillapex and BioRoot RCS, set more quickly, reducing prolonged exposure to harmful substances [7].

AH Plus (Dentsply DeTrey, Konstanz, Germany) is a hydrophobic epoxy resin sealer recognized for its exceptional long-term sealing capabilities, minimal dimensional changes, and high radiopacity. These attributes have led to its widespread adoption in clinical practice, establishing it as the gold standard in root canal sealing [8,9]. Nonetheless, certain drawbacks should be recognized, such as challenges in achieving a reliable bond with gutta-percha, questionable adhesion to canal walls when moisture is present, and complications during removal in retreatment scenarios [10]. One major concern is the potential cytotoxicity and genotoxicity of epoxy resin sealers, including AH Plus and AH 26. Research has demonstrated that these sealers exhibit cytotoxic effects, particularly in unsealed conditions and at higher concentrations in vitro, especially in fibroblasts [5,11,12,13]. Bioceramic sealers, formulated from tricalcium silicate endodontic cement, are commonly referred to as mineral trioxide aggregate (MTA)-based sealers. These hydrophilic materials require humidity for setting, and exhibit excellent biocompatibility and a strong ability to form hydroxyapatite, promoting favorable outcomes in endodontic procedures [14]. Bioceramic sealers, such as TotalFill BC Sealer, demonstrate clear advantages over epoxy resin sealers like AH Plus in endodontics. These bioceramic materials, particularly TotalFill BC Sealer, show significantly higher push-out bond strength compared to epoxy resin sealers such as AH Plus [15]. Additionally, bioceramic sealers exhibit better adaptation to root canal walls, effectively reducing microleakage across various clinical scenarios [16]. Regarding bond strength, while AH Plus may exhibit higher push-out bond strength in some instances, bioceramic sealers, such as BioRoot RCS, perform comparably and provide additional bioactive properties that promote hard tissue formation. This makes them particularly suitable for clinical applications where biointegration is essential [17].

Efforts to determine the most biocompatible calcium silicate sealer remain inconclusive. While BioRoot RCS is generally more biocompatible than alternatives like iRoot SP, MTA Fillapex, and Endoseal MTA, this conclusion is limited by the available literature [18,19,20,21,22]. Despite similar chemical compositions, these sealers show varying degrees of cytocompatibility, influenced by differences in commercial formulations, including unknown fillers and thickening agents [23]. Among them, MTA Fillapex is identified as having the lowest biocompatibility, exhibiting significant cytotoxicity, largely due to its salicylate resin component, prolonged setting time, and toxic leaching [18,19,20,21,24,25]. Additionally, it has higher solubility than AH Plus, even after setting [26]. However, a recent study suggested improved cell attachment and proliferation with MTA Fillapex, possibly due to a reformulation substituting calcium tungstate for bismuth oxide [23].

Previous in vitro studies have compared the cytotoxicity and genotoxicity of bioceramic and epoxy resin sealers, utilizing various cell types to assess their biological impact. Research has been conducted using human periodontal-like cells and periodontal fibroblasts [27,28,29,30,31], osteoblasts [32,33,34,35], and gingival fibroblasts [18,23,24,36,37,38]. The findings of these studies underscore the potential of premixed calcium silicate-based root canal sealers as a more biocompatible alternative to AH Plus, suggesting their advantageous application in endodontic treatments [38].

Based on the context and previous knowledge of bioceramic sealers in endodontics, their routine use in dental practice, and contradictory results stemming from studies with varying findings—with some showing no significant differences between materials, and others reporting superior properties for bioceramic sealers, while some indicate the opposite [3,5,11,12,18,19,20,21,22,24,27,28,29,30,31,32,33,34,35,36,37,38]—the main objective of this in vitro study was to evaluate the cytotoxic and genotoxic effects of calcium silicate-based sealers (BioRoot RCS and MTA Fillapex), in comparison with the epoxy resin-based sealer AH Plus, using two cell lines, HepG2 and V79, and employing the micronucleus and comet assays. It was hypothesized that calcium silicate-based sealers would have lower effects than AH Plus, with greater toxicity at higher concentrations.

## 2. Materials and Methods

### 2.1. Preparation of Materials and Extraction of Non-Reacted Components

Three different root canal sealers were tested in this study: one epoxy resin-based sealer (AH Plus) and two calcium silicate-based sealers (MTA Fillapex and BioRoot RCS). The compositions of the tested materials are listed in Table 1.

**Figure 1:**
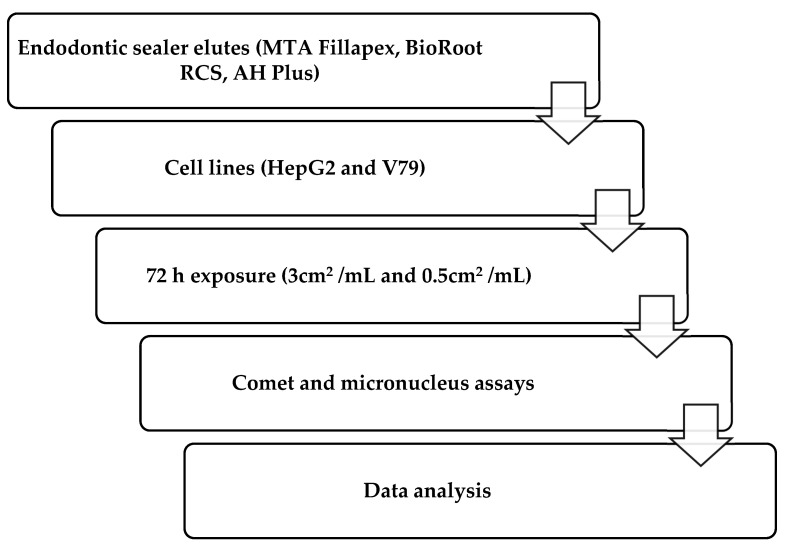
Flowchart of study design and methodology.

In compliance with the norm ISO 10993-12 (sample preparation and reference materials), molds were designed which were used to obtain composite samples with the required surface area of 3 cm^2^ (0.6 cm × 2 cm × 0.1 cm) [39]. An eluate of 3 cm^2^/mL of medium was required for the treatment. For both tested concentrations, duplicate slides were prepared and analyzed in five independent experiments. Components of the materials were mixed and left to set under the sterile conditions at 37 °C, according to the manufacturers’ instructions, in a humid chamber at 37 °C. Afterward, materials were introduced into the cell culture medium for HepG2 cells (Eagle’s Minimum Essential Medium—EMEM) or into Dulbecco’s Modified Eagle Medium (DMEM) for treatment of V79 cells. The elution time was 72 h. The study procedure is illustrated in the flowchart presented in Figure 1.

### 2.2. Cell Culture

The cytotoxic and genotoxic effects of the residual components of the composites were tested on two cell lines—a Chinese hamster lung fibroblast V79 cell line and a human hepatocellular carcinoma HepG2 cell line. We decided to use a HepG2 cell line since it possesses an endogenous metabolic activation system, allowing us to investigate both the effects of the compounds in their paternal state, and, if there was one, in their metabolically changed form [40,41]. V79 cells are cells originating from healthy fibroblasts, and will indicate cyto/genotoxic-only paternal molecules of chemicals released from dental materials [42]. Both the V79 and HepG2 cell lines were obtained from ATTC (Glasgow, UK). After thawing them, they were incubated in TPP tissue culture flasks with a surface area of 25 cm^2^ (TPP, Trasadingen, Switzerland), with filtered caps. Concerning the HepG2 cell line, cells were cultured in 5 mL of EMEM, while V79 cells were cultured in mL of DMEM. Both cell cultures were supplemented with 1% (*v*/*v*) fetal bovine serum (Merck Sigma Aldrich, Taufkirchen, Germany) and 0.1 mL of penicillin/streptomycin (100 IU/mL or 100 µg/mL final concentration, respectively; Merck Sigma Aldrich). The cells were left to grow without the presence of the sealer eluate for 48 h, in an atmosphere of 5% CO_2_ at 37 °C. Once they covered the bottom surface, they were detached from the flask using trypsin 0.25% (*v*/*v*) in EDTA (Merck Sigma Aldrich). They were centrifuged, the eluate was removed, and the pellet was resuspended in a fresh medium with the addition of the eluate of the sealer. Following the second cell collection, the cells were seeded in a Nunc^®^ Lab-Tek^®^ Chamber Slide™ system and treated with 1 mL of the eluates obtained from the materials (3 cm^2^/mL). Each material was exposed to two volumes of eluates—the highest one in compliance with the norm ISO 10993-12, and second corresponding to the amount of the material implemented in the clinical procedures (0.5 cm^2^/mL). Both the HepG2 and V79 cell lines were cultivated and manipulated in the same manner. The cell lines were treated for 72 h in an atmosphere of 5% CO_2_, at 37 °C. No change in the color of the medium was observed, which indicated that the pH of the medium was not changed by the addition of treatment eluate. For the alkaline comet assay analysis, the cells were harvested immediately after the treatment ended. As positive controls, for the HepG2 cell line, cyclophosphamide in a final concentration of 300 µg/mL was added to the culture 72 h prior to harvesting. The substance was used as a reference to determine the efficiency of the experimental system by recording the genotoxic effect of the metabolites in the experimental system applied. For the V79 cell line, as a positive control, ethyl methanesulfonate at a concentration of 25 mg/mL was added simultaneously with cytochalasin B. Negative controls were vehicle controls, e.g., 100 µL of the cell culture medium.

### 2.3. Alkaline Comet Assay

Following 72 h of treatment, the medium was removed, and cells were washed 3 times with saline solution. Trypsin in EDTA was added to detach the cells from the cell culture flask and from each other. The cells were centrifuged, the supernatant was removed, and the pellet was resuspended in 1 mL of fetal bovine serum and 4 mL of cell culture medium to inhibit trypsin’s activity. The cells were centrifuged twice and resuspended in saline solution. Following the last centrifugation, the cells were suspended in a sufficient volume of solution to obtain 107 cells/mL. On microscope slides previously coated with a 1% (Merck Sigma Aldrich) normal-melting-temperature agarose *w*/*v* (NMP), cells were resuspended in 5% m/v low-melting-point agarose (LMA). A 10 mL volume of cells was resuspended in 110 mL of LMA. The suspension was placed on the precoated microscopic slide, covered with a 24 × 40 mm cover slip, set on ice, and allowed to polymerize. The cover slide was removed, and preparations were submersed in chilled lysis solution (100 mmol/L Na_2_EDTA, 2.5 mol/L NaCl, 1% Na-laurylsarcosinate, 10 mmol/L Tris-HCl, 10% dimethyl sulfoxide, and 1% Triton X-100; pH = 10 + 4 °C, pH > 13) for 20 min. The preparations were transferred to a denaturation buffer containing 300 mM NaOH and 1 mM Na_2_EDTA, +4 °C, with a pH > 13. Electrophoresis was performed in the same buffer as used for the denaturation. The time of electrophoresis was 20 min at 300 mA and 0.85 mV/cm. Following the electrophoresis, the preparations were rinsed in redistilled water and stained with ethydium bromide 20 µg/mL. The slides were analyzed using an epifluorescent microscope, in accordance with the scoring criteria set in the OECD’s 2016 guidelines [43]. For each treatment, 100 nucleoids were analyzed under the epifluorescent microscope Olympus B50 (Tokyo, Japan) connected with a live CCD camera to the Comet Assay IV (version 4.3) (Instem-Perceptive Instruments, Suffolk, UK) software. For every nucleoid, the tail length (µm) and tail intensity (% DNA in tail) were measured under 200× magnification. Hedgehogs, nucleoids with small heads and more than 80% of their DNA in the tail, were excluded from the analysis.

### 2.4. Micronucleus Cytome Assay

Following the treatment, all the media were removed from the chambers and the cell cultures were treated with 10 M KOH for 10 min. Afterwards, the solution was discarded, and a fixative (acetic acid and methanol 1:3 *v*/*v*) was added for 5 min. The fixation process was repeated 4 times until either the pellet or the supernatant became colorless. The margins of the cover were removed, and after being air-dried, the slides were subjected to staining with 5% Giemsa.

The micronucleus cytome assay enabled us to assess cytotoxicity, along with genotoxicity. For each treatment, 2000 binucleate cells were scored for the presence of a micronucleus, a nuclear bud, a nucleoplsmic bridge, apoptosis, or necrosis, in compliance with the protocol by Fenech (2007) [43]. The analysis was performed under the light microscope Olympus CX40 (Tokyo, Japan) at 600× magnification.

To determine whether the eluates affected the dynamics of the cell cycle (cryostasis or proproliferative effects), the Cytokinesis-Block Proliferation Index (CBPI) was determined by scoring the ratio of mononuclear, binucleate, three-nucleate, four-nucleate, and oligonucleate cells in 1000 cells per treatment. The CBPI was determined according to the following formula [44]:

$$\mathrm{CBPI}=\frac{No.\;of\;mononuceate+2\times No.\;of\;binucleate+3\times (No.\;of\;three,four\;and\;oligonucleate\;cells)}{1000(No.of\;total\;scored\;cells)}$$

### 2.5. Data Analysis

To evaluate the normality of the data distribution, the Kolmogorov–Smirnov test was applied. Descriptive statistics, including means and standard deviations for continuous variables, were used to provide a summary of the data. The relationships between the two concentrations of the sealers were analyzed using the Mann–Whitney test, while differences among materials and between positive and negative controls were assessed using the Kruskal–Wallis test with post hoc analysis. Statistical significance was set at *p* < 0.05, and the data were analyzed using SPSS Version 26 (IBM Corp, Armonk, NY, USA).

## 3. Results

**Figure 2:**
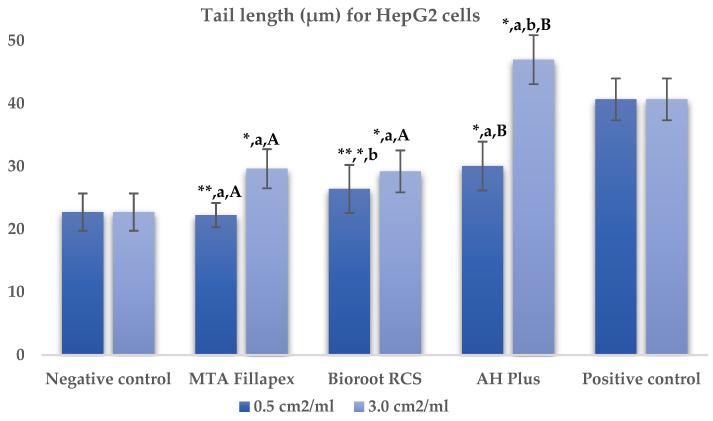
Bar graphs presenting the tail length (μm) results for HepG2 cell lines after exposure to different materials at varying concentrations. Symbols: Identical lowercase letters within the same concentration indicate statistically significant differences between different materials, while * denotes a statistically significant difference compared to the negative control, and ** signifies a statistically significant difference compared to the positive control. Additionally, identical uppercase letters indicate statistically significant differences within the same material across different concentrations.

**Figure 3:**
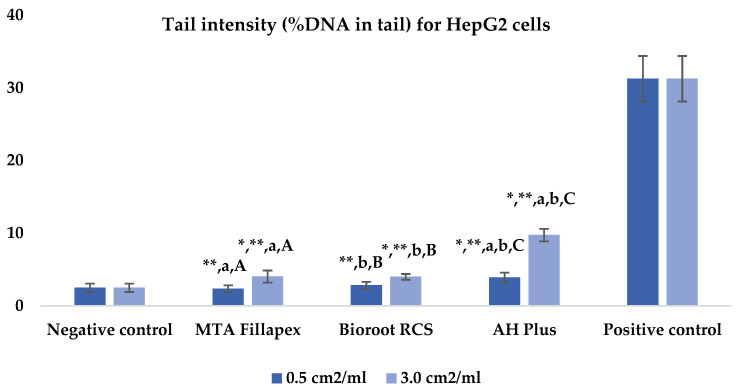
Bar graphs presenting the tail intensity (%DNA in tail) results for HepG2 cell lines after exposure to different materials at varying concentrations. Symbols: Identical lowercase letters within the same concentration indicate statistically significant differences between different materials, while * denotes a statistically significant difference compared to the negative control, and ** signifies a statistically significant difference compared to the positive control. Additionally, identical uppercase letters indicate statistically significant differences within the same material across different concentrations.

Table 2, Figure 2 and Figure 3 presents the results of the comet assay for the HepG2 cell line. Higher concentrations of all three materials led to increased genotoxic effects, with significant differences observed between the concentrations (0.5 cm^2^/mL vs. 3.0 cm^2^/mL, *p* < 0.05) for both measured parameters (tail length and intensity), except for tail length with the BioRoot RCS sealer (*p* = 0.190). MTA Fillapex showed significantly lower values (*p* ≤ 0.001) for both comet assay parameters compared to AH Plus at all concentrations. BioRoot RCS also displayed lower tail intensity at both concentrations compared to AH Plus, but only showed reduced tail length at the higher concentration (*p* ≤ 0.001). Although BioRoot RCS had higher values for both parameters compared to MTA Fillapex, no statistically significant difference was observed between these two materials (*p* > 0.05). In contrast to the AH Plus endodontic sealer, MTA Fillapex and BioRoot RCS showed a statistically significant difference in tail length compared to the positive control at the lower concentration (*p* ≤ 0.001). However, at the lower concentration, neither of the two tested bioceramic materials showed a statistically significant difference compared to the negative control for tail length and tail intensity, with MTA Fillapex even showing lower values (*p* > 0.05).

**Figure 4:**
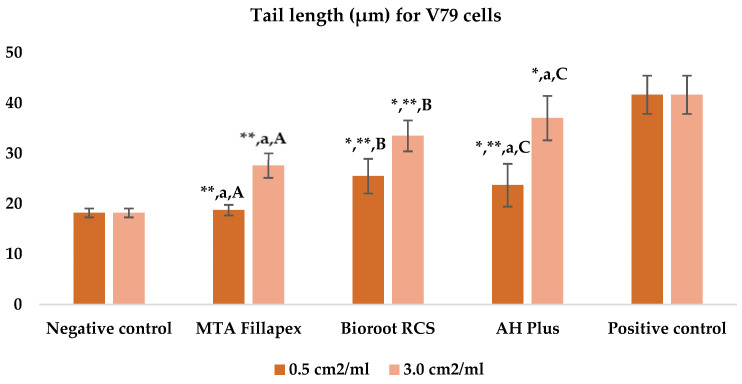
Bar graphs presenting the tail length (μm) results for V79 cell lines after exposure to different materials at varying concentrations. Symbols: Identical lowercase letters within the same concentration indicate statistically significant differences between different materials, while * denotes a statistically significant difference compared to the negative control, and ** signifies a statistically significant difference compared to the positive control. Additionally, identical uppercase letters indicate statistically significant differences within the same material across different concentrations.

**Figure 5:**
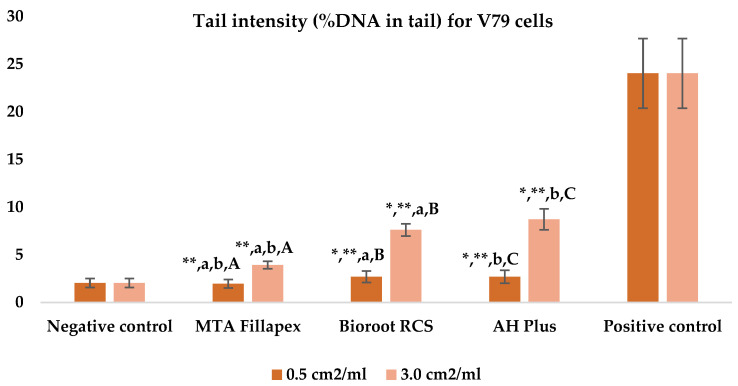
Bar graphs presenting the tail intensity (%DNA in tail) results for V79 cell lines after exposure to different materials at varying concentrations. Symbols: Identical lowercase letters within the same concentration indicate statistically significant differences between different materials, while * denotes a statistically significant difference compared to the negative control, and ** signifies a statistically significant difference compared to the positive control. Additionally, identical uppercase letters indicate statistically significant differences within the same material across different concentrations.

Table 3, Figure 4 and Figure 5 shows the results of the comet assay for the V79 cell line. Like the HepG2 cell line, higher concentrations of all three of the sealers tested led to increased genotoxic effects, with significant differences observed between different concentrations for both parameters measured. MTA Fillapex showed significantly lower values (*p* ≤ 0.001) for both comet assay parameters compared to AH Plus at both concentrations (*p* ≤ 0.001). Although BioRoot RCS caused less damage than AH Plus for both tested comet assay parameters at both concentrations, the difference was not statistically significant. Also, while BioRoot RCS had higher values for both parameters compared to MTA Fillapex, no statistically significant difference was found for tail length (*p* > 0.05), but there was a significant difference for tail intensity at both concentrations (*p* ≤ 0.001). MTA Fillapex showed no statistically significant differences at both concentrations and for both parameters tested compared to the negative control (*p* > 0.05).

Table 4 represents the results of the micronucleus cytome assay on the HepG2 cell line. Compared to AH Plus, MTA Fillapex caused less cytogenetic damage, with fewer micronuclei and nuclear buds and a higher number of cells with undamaged chromatin at the lower concentration (*p* < 0.05). At the higher concentration, MTA Fillapex had a lower number of micronuclei, but a higher Cytokinesis-Block Proliferation Index (CBPI). Similarly, at the lower concentration, BioRoot RCS also caused less cytogenetic damage than AH Plus, with fewer micronuclei and nuclear buds, and a higher number of cells with undamaged chromatin. However, at the higher concentration, BioRoot RCS showed an increase in nuclear buds and CBPI, while the number of cells with undamaged chromatin remained higher (*p* < 0.05). Between the two bioceramic sealers, BioRoot RCS induced fewer nuclear buds and nucleoplasmic bridges than MTA Fillapex at the lower concentration, and fewer nuclear buds alone at the higher concentration. At lower concentrations, MTA Fillapex induced a significantly increased number of nucleoplasmic bridges compared to the negative control, while at both concentrations, it caused apoptosis.

Table 5 shows the results of the micronucleus cytome assay for the V79 cell line. MTA Fillapex, compared to BioRoot RCS, resulted in a significantly lower count of nuclear buds and a higher CBPI. In comparison with AH Plus, MTA Fillapex showed a significantly lower number of micronuclei and more cells with undamaged chromatin, while BioRoot RCS, compared to AH Plus, resulted in a significantly lower count of micronuclei, nuclear buds, and apoptotic cells, as well as a higher count of cells with undamaged chromatin and a higher CBPI (*p* < 0.05).

## 4. Discussion

In the field of endodontics, the choice of sealing materials is crucial for ensuring successful treatment outcomes and minimizing risks related to cytotoxicity, inflammatory response, and long-term biocompatibility [1,3]. This study aimed to evaluate the cytotoxic and genotoxic effects of calcium silicate-based sealers (BioRoot RCS and MTA Fillapex) compared to an epoxy resin-based sealer (AH Plus). The hypothesis that calcium silicate-based sealers would have lower cytotoxic and genotoxic effects than AH Plus was confirmed by the results of this study. Both MTA Fillapex and BioRoot RCS showed less cytotoxic and genotoxic damage in terms of the tested micronucleus and comet assay parameters compared to AH Plus in both cell lines. These findings are consistent with most of the literature, which indicates greater cytotoxicity and genotoxicity of epoxy resin-based sealers compared to calcium silicate-based sealers [19,27,45,46]. However, it is important to acknowledge the existence of contradictory results, as some studies have reported no statistically significant differences in cytotoxicity or biological effects between these two types of sealers [30,47].

The importance of the comet assay lies in its sensitivity and precision in detecting DNA damage at the level of individual cells, making it a valuable tool for assessing genotoxicity in various materials. Genotoxicity is a crucial factor in evaluating the potential risks of materials in direct contact with tissues. Studies on genotoxicity, including those employing the comet assay, offer valuable insights into genetic damage that may contribute to carcinogenesis or other long-term health effects. Research using the comet assay has been employed to evaluate the genotoxicity of bioceramic sealers, underscoring its significance in assessing the safety of endodontic materials. Additionally, these findings align with regulatory guidelines that highlight genotoxicity as a crucial parameter in material safety assessments [5,48,49]. In this study, MTA Fillapex showed no significant primary damage to DNA (measured as nucleoids’ tail length and intensity) at lower concentrations compared to the negative control in both of the cell lines tested. At higher concentrations (eluates of 3 cm^2^), however, a significant increase in the primary damage induced in the HepG2 cell line compared to the negative control was observed. Conversely, BioRoot RCS showed slightly greater DNA damage in both cell cultures and for both comet assay parameters, indicating that leached unreacted components can induce primary damage to DNA. Similarly, in a related study that used periodontal ligament cells with lentiviral hTERT gene transfer and the γH2AX immunocytochemistry assay, the MTA Fillapex sealer also showed no significant difference compared to the negative control. However, BioRoot RCS, in that study, demonstrated an increase, though statistically insignificant, in DNA double-strand breaks in PDL-hTERT cells compared to the negative control [19]. Furthermore, in an in vitro study investigating the genotoxicity of endodontic sealers using the comet assay, it was found that the gingival fibroblast cell line showed statistically significantly higher DNA damage for BioRoot RCS and AH Plus eluates compared to the negative control, both 24 and 48 h after incubation. Additionally, in a peripheral blood monocyte/macrophage cell line, DNA damage was also statistically significantly greater for BioRoot RCS and AH Plus eluates. After 48 h, both sealers showed statistically higher DNA damage compared to the negative control [47]. In the present study, the AH Plus sealer showed a statistically significant difference compared to the negative control in both cell lines, for both concentrations, in both parameters of the comet test. The genotoxicity of AH Plus has already been confirmed in various cell lines [5,12,50]. In an in vitro study on L929 mouse fibroblasts, the genotoxicity of epoxy resin-based root canal sealers was investigated using the comet assay. The results confirmed an increase in DNA damage by the AH Plus sealer compared to the negative control, although this increase was only statistically significant in the unsealed form [5].

When comparing the tested bioceramic sealers with the epoxy-based AH Plus sealer, the comet assay revealed that MTA Fillapex exhibited significantly lower genotoxicity in both cell lines and at both tested concentrations. BioRoot RCS also showed significantly lower tail intensity in HepG2 cells compared to AH Plus. These findings are consistent with previous in vitro studies on human gingival fibroblasts, where MTA Fillapex showed minimal genotoxic effects, even at high concentrations [51]. Supporting our results, a study on mouse fibroblasts confirmed the genotoxicity of AH Plus, with both set and unset samples increasing tail intensity in comet assays [5]. However, contradictory data exist. An in vitro study on human periodontal ligament fibroblasts found lower tail intensity and a shorter tail length for AH Plus compared to MTA Fillapex [46]. Similarly, studies using CHO-K1 cells have reported stronger genotoxicity for MTA Fillapex than AH Plus, based on comet and γH2AX immunofluorescence assays [52]. Additionally, results from human lymphocytes showed slightly higher γH2AX expression in the resin-based sealer group, suggesting a marginally stronger genotoxic response than in the bioceramic group [53].

It is critical to note that the AH Plus root canal sealer contains bisphenol A (BPA), a potent endocrine-disrupting compound, integrated as a monomer in dental resins. BPA is present in various analogs and derivatives, and its leaching from resin-based materials may induce toxic effects, disrupting endocrine function and hormonal regulation [54]. However, this substance may lead to cross-linking reactions that can affect the results of the comet assay by potentially reducing the visible DNA damage, making it less detectable. This phenomenon occurs due to the cross-linking properties of bisphenol compounds, which can create complex molecular structures that interfere with DNA migration in the assay, thus impacting genotoxicity assessment accuracy [55]. Another molecule present in AH Plus sealer is Bis-GMA, recognized as a genotoxic, clastogenic, and aneugenic compound, which is small and able to penetrate the site of application and affect the surrounding cells and macromolecules, specifically DNA [56]. Therefore, further investigation into the effects of these sealers on DNA integrity and their potential genotoxicity is essential for improving safety standards in endodontic materials.

In contrast to the HepG2 cell line, where no significant difference was observed between the two bioceramic endodontic sealers tested, in the V79 cell line, BioRoot RCS induced greater tail intensity (%DNA in tail) compared to MTA Fillapex at both concentrations. The results of this study may be attributed to the fact that BioRoot RCS is mixed manually, which can lead to deviations in the powder-to-liquid ratio and disruptions in crystallization. This is an important limitation that should be considered, as it may affect the outcomes of the genotoxicity study [57]. Our results can be supported by the findings of an in vitro study in which the cytocompatibility of MTA Fillapex and BioRoot RCS was investigated on human gingival fibroblasts using an indirect test method. The results indicated that the BioRoot RCS eluent was toxic to gingival fibroblasts in both the 1-day and 28-day eluates, while MTA Fillapex showed optimal cell activity in the 1-day eluent, which deteriorated in the 28-day eluent. This suggests that MTA Fillapex does not initially release toxic substances, but toxic effects may occur over time [23]. Our results, along with theirs, are somewhat inconsistent with the majority of biocompatibility studies on these materials, where BioRoot RCS is generally considered less toxic than MTA Fillapex [58,59,60]. This confirms the results of an in vivo biocompatibility study of BioRoot RCS, MTA Fillapex, and AH Plus, in which freshly mixed materials were implanted into the subcutaneous tissue of Wistar rats; the rats showed a significant reduction in inflammatory response over 7, 14, 30, and 60 days, with complete healing observed, in addition to thinning of the fibrous capsule, confirming the biocompatibility of all three sealers [61].

The micronucleus test is crucial for evaluating endodontic materials, as it assesses their genotoxic potential, ensuring safety for surrounding tissues. It provides regulatory compliance, facilitates comparative analysis of different sealers, and helps to anticipate long-term health risks, thereby safeguarding patient health against potential DNA damage and carcinogenic effects [27,36,46,52,62]. The micronucleus assay conducted in this study identified potential genotoxic and cytotoxic effects associated with the examined endodontic sealers. In both cell lines, MTA Fillapex, BioRoot RCS, and AH Plus induced a significantly higher number of apoptotic cells compared to the negative control. Additionally, AH Plus exhibited an increased incidence of necrotic cells, micronuclei formation, nuclear buds, and nucleoplasmic bridges, alongside reduced CBPI levels in the HepG2 cell line, highlighting the pronounced cytotoxic potential of this epoxy resin-based sealer. Compared to the bioceramic sealers, the AH Plus sealer induced markedly higher cytogenetic damage than both MTA Fillapex and BioRoot RCS, as indicated by a significantly greater number of micronuclei in both cell lines. When comparing the two bioceramic sealers, BioRoot RCS demonstrated fewer nuclear buds across both tested concentrations in the HepG2 cell line, indicating a potentially lower genotoxic impact than MTA Fillapex. In contrast, MTA Fillapex showed reduced nuclear bud formation, alongside an elevated CBPI, in the V79 cell line, suggesting variable genotoxic responses between cell types. Similar observations have been reported in other studies. According to XTT-based cell viability assays, the epoxy resin-based sealer AH Plus exhibited higher cytotoxicity in periodontal ligament cell lines compared to MTA Fillapex and BioRoot RCS [19]. Additionally, micronucleus assays in other studies have revealed genotoxic effects of AH Plus sealers, shown by an increased number of micronuclei in human gingival fibroblasts and human periodontal ligament fibroblasts compared to other bioceramic root canal sealers [27,36]. Consistent with the findings of this study, AH Plus also induced a significant number of micronuclei in the V79 cell line [63]. Conversely, one study reported no statistically significant differences in micronuclei counts across these sealers in a human periodontal ligament stem cell line, while also noting that MTA Fillapex demonstrated higher cell viability compared to the AH Plus sealer [30]. However, some studies have reported different findings regarding the comparison of cyto- and genotoxicity between epoxy resin and bioceramic sealers. An in vitro study on the V79 cell line showed that both AH Plus and MTA Fillapex led to increased micronucleus formation compared to the control, with MTA Fillapex identified as the most genotoxic material [62].

MTA Fillapex exhibits physical–chemical properties that align more closely with those of resin-based sealers than with those of hydraulic sealers. As such, it retains certain biological characteristics associated with hydraulic cement, including alkalinization and hydroxyapatite precipitation, while demonstrating markedly lower biocompatibility relative to calcium silicate-based sealers [19,27,45,46]. However, several studies have documented that MTA Fillapex exhibits greater cytotoxicity than AH Plus materials [18,45,49,60,61,62,63,64]. The higher genotoxicity of MTA Fillapex might be due to the bismuth in its composition, which is a transition metal, and by Fenton and Haber Weiss-related reactions, it might damage macromolecules in the cells exposed to it, including DNA [65]. The cytotoxic properties of the MTA Fillapex sealer may also be attributed to its salicylate resin component, which is associated with prolonged setting times and leaching of material [25].

The potential genotoxicity and cytotoxicity of certain sealers may be linked to their setting time and the release of components during or after curing [1,6,7]. For epoxy-based sealers like AH Plus, which has a setting time of around 8 h, factors such as the resin-to-hardener ratio, the elapsed time after mixing, and environmental surface properties can influence the release of extractable monomers. These include formaldehyde and other organic or inorganic compounds known to cause local and systemic toxicity, particularly affecting periradicular tissues and alveolar bone [5]. Their leaching during polymerization may trigger inflammation and cell damage. In contrast, bioceramic sealers like MTA Fillapex and BioRoot RCS set faster (approximately 2.5 and 4 h, respectively) [1,6], but may still release irritants such as calcium hydroxide and silicon ions, which can cause local tissue irritation and interfere with healing [64].

The elution time of materials used in endodontics, such as epoxy resin and bioceramic sealers, can significantly impact their toxicity. Cytotoxicity is highest immediately after the material is applied and before it sets, and it decreases 24 h after it has set, with continued reduction over time as it hardens [66]. Few studies have investigated the genotoxicity of endodontic sealers using the comet assay at different elution times, and contradictory results have been obtained. A study on periodontal fibroblasts confirmed a decrease in the intensity and length of the tail of AH Plus and MTA Fillapex after 48 h compared to after 24 h [49], while in a study examining genotoxicity to human fibroblasts, the BioRoot RCS sealer showed increased DNA damage (tail intensity) in the comet assay after 48 h compared to after the 24 h elution [47].

This study has several limitations. The genotoxicity assessment of hydraulic and epoxy-based sealers may be influenced by variables such as setting time (set vs. unset material), mixing time, moisture requirements (especially for bioceramics), and dilution protocols. These factors can significantly affect material behavior and cellular response. Moreover, the absence of multiple evaluation time points, as highlighted in other studies, limits toxicity assessment over time (e.g., 24 h, 48 h, 7 days, 14 days). In addition to genotoxicity, evaluating cytotoxicity through tests like the micronucleus assay or cell viability assays would have provided a more complete toxicological profile. The in vitro nature of this study also limits direct clinical applicability, as it reflects isolated cellular responses [67]. Blood flow models might better replicate the in vivo environment by accounting for vascular effects on material toxicity. Furthermore, the experimental design does not fully mimic clinical conditions—variations in contact area, volume, and operator technique may increase tissue exposure. In vitro genotoxicity tests lack the systemic complexity of an organism, including metabolic functions that can activate or neutralize compounds, some of which become genotoxic only after hepatic metabolism. Short exposure periods may miss cumulative or chronic effects. Additionally, interspecies variability, the absence of immune responses, and a lack of tissue interactions reduce extrapolability to clinical contexts [68]. Future studies should integrate both in vitro and in vivo approaches. Advances in 3D cell cultures and organoid models could better replicate tissue dynamics. Human cell lines combined with metabolic systems simulating liver function would enhance genotoxicity accuracy. Long-term exposure studies and in vivo models targeting tissues directly exposed to sealers could offer further insights into potential genotoxic risks [69]. Recent studies employing in vivo genetic analyses have provided valuable insights into the evaluation of sealer biocompatibility and biological effects. One such study, despite focusing on different types of sealers, utilized advanced genetic techniques to assess the interaction between root canal sealers and host tissue. By examining gene expression profiles, the researchers were able to evaluate inflammatory and genotoxic responses to the materials over time. This approach offers a more comprehensive understanding of how endodontic materials, including sealers, affect cellular mechanisms and tissue responses in vivo, thus providing a relevant methodological framework for future biocompatibility studies [70].

## 5. Conclusions

In conclusion, this study demonstrated that both MTA Fillapex and BioRoot RCS exhibited a more favorable cytotoxic and genotoxic profile compared to AH Plus, with MTA Fillapex showing the lowest genotoxicity overall. BioRoot RCS also showed lower genotoxicity than AH Plus, though it had slightly higher values than MTA Fillapex. Clinically, these results suggest that both MTA Fillapex and BioRoot RCS may be safer alternatives for use in endodontic procedures.

## Figures and Tables

**Table 1:** The composition of the root canal sealers used in the study.

Sealer	Manufacturer	Type	Composition	Presentation	LOT
MTA Fillapex	Angelus, Londrina, PR, Brazil	Calcium silicate-based sealer	Paste A: salicylate resin, bismuth trioxide, fumed silica Paste B: fumed silica, titanium dioxide, mineral trioxide aggregate, base resin	Two tubes	71695
BioRoot RCS	Septodont, Saint Maur Des Fosses, France	Hydraulic calcium silicate-based sealer	Powder: tricalcium silicate, zirconium oxide, povidone Liquid: aqueous solution of calcium chloride and polycarboxylate	Powder/liquid	B30723C B31424
AH Plus	Denstply DeTrey GmbH, Konstanz, Germany	Epoxy-based sealer	Paste A: diepoxide, calcium tungstate, zirconium oxide, aerosil, pigments Paste B: 1-adamantane amine, N,N’-dibenzyl-5-oxa-nonandiamine-1,9 TCD-diamine, calcium tungstate, zirconium oxide, aerosil, silicone oil	Two tubes	2207000721

**Table 2:** Comet alkaline assay results for HepG2 cell line after exposure to different materials at varying concentrations.

Material	Tail Length (μm)		*p*-Value ^†^	Tail Intensity (%DNA in Tail)		*p*-Value ^†^
	0.5 cm^2^/mL	3.0 cm^2^/mL		0.5 cm^2^/mL	3.0 cm^2^/mL	
Negative control	22.74 ± 2.96		n. a.	2.49 ± 0.58		n. a.
MTA Fillapex	22.26 ± 1.73 **^,a^	29.64 ± 3.12 *^,a^	≤0.001	2.38 ± 0.45 **^,a^	4.04 ± 0.83 *^,^**^,a^	≤0.001
BioRoot RCS	26.43 ± 3.82 **	29.22 ± 3.33 *^,b^	0.190	2.83 ± 0.48 **^,b^	4.00 ± 0.40 *^,^**^,b^	≤0.001
AH Plus	30.06 ± 3.88 *^,a^	46.98 ± 3.91 *^,a,b^	≤0.001	3.90 ± 0.62 *^,^**^,a,b^	9.75 ± 0.86 *^,^**^, a,b^	≤0.001
Positive control	40.67 ± 3.31		n. a.	31.25 ± 3.13		n. a.
*p*-value ^††^	≤0.001	≤0.001		≤0.001	≤0.001	

The data are presented as the mean and standard deviation. ^†^ Mann–Whitney test; ^††^ Kruskal–Wallis test; *p* < 0.05. In the columns, the same lowercase letter in superscript indicates a difference between the materials. Symbols: * statistically significant compared to the negative control; **, statistically significant compared to the positive control; n. a., not acceptable.

**Table 3:** Comet alkaline assay results for V79 cell line after exposure to different materials at varying concentrations.

Material	Tail Length (μm)		*p*-Value ^†^	Tail Intensity (%DNA in Tail)		*p*-Value ^†^
	0.5 cm^2^/mL	3.0 cm^2^/mL		0.5 cm^2^/mL	3.0 cm^2^/mL	
Negative control	18.20 ± 0.88		n. a.	2.04 ± 0.48		n. a.
MTA Fillapex	18.74 ± 1.06 **^,a^	27.63 ± 2.46 **^,a^	≤0.001	1.96 ± 0.45 **^,a,b^	3.93 ± 0.39 **^,a,b^	≤0.001
BioRoot RCS	25.31 ± 3.44 *^,^**	33.51 ± 3.07 *^,^**	≤0.001	2.69 ± 0.60 *^,^**^,a^	7.61 ± 0.64 *^,^**^,a^	≤0.001
AH Plus	23.71 ± 4.26 *^,^**^,a^	37.05 ± 4.41 *^,a^	≤0.001	2.70 ± 0.68 *^,^**^,b^	8.73 ± 1.10 *^,^**^,b^	≤0.001
Positive control	41.67 ± 3.78		n. a.	24.40 ± 3.66		n. a.
*p*-value ^††^	≤0.001	≤0.001		≤0.001	≤0.001	

The data are presented as the mean and standard deviation. ^†^ Mann–Whitney test; ^††^ Kruskal–Wallis test; *p* < 0.05. In the columns, the same lowercase letter in superscript indicates a difference between the materials. Symbols: *, statistically significant compared to the negative control; **, statistically significant compared to the positive control; n. a., not acceptable.

**Table 4:** Micronucleus cytome assay results for HepG2 cell line after exposure to different materials at varying concentrations.

Cytogenetic Damage	Concentration	Negative Control	MTA Fillapex	Bioroot RCS	AH Plus	Positive Control	*p*-Value ^††^
Micronucleus	0.5 cm^2^/mL	7.50 ± 1.90	7.40 ± 2.84 **^,a^	7.30 ± 2.87 **^,b^	16.90 ± 4.53 *^,a,b^	46.40 ± 3.41	≤0.001
	3.0 cm^2^/mL		7.10 ± 2.42 **^,a^	11.60 ± 2.91 **	21.40 ± 3.81 *^,a^		≤0.001
*p*-value ^†^		n. a.	0.853	0.005	0.023	n. a.	
Nuclear buds	0.5 cm^2^/mL	20.18 ± 5.15	21.50 ± 6.59 **^,a,b^	14.40 ± 2.84 **^,a,c^	31.90 ± 4.95 *^,b,c^	45.40 ± 4.13	≤0.001
	3.0 cm^2^/mL		39.80 ± 5.12 *^,a^	24.90 ± 4.58 **^,a,b^	42.30 ± 6.78 *^,b^		≤0.001
*p*-value ^†^		n.a.	≤0.001	≤0.001	0.002	n. a.	
Nucleoplasmic bridges	0.5 cm^2^/mL	1.60 ± 1.51	3.80 ± 1.48 *^,a^	1.10 ± 0.74 **^,a^	2.80 ± 2.78 *^,^**	8.30 ± 2.34	≤0.001
	3.0 cm^2^/mL		2.30 ± 1.25 **	2.70 ± 1.42 **	4.30 ± 2.45 *^,^**		≤0.001
*p*-value ^†^		n. a.	0.043	0.007	0.190		
CBPI	0.5 cm^2^/mL	1.84 ± 0.07	1.86 ± 0.07 **^,a^	1.84 ± 0.05	1.76 ± 0.07 **^,a^	1.64 ± 0.04	≤0.001
	3.0 cm^2^/mL		1.77 ± 0.09 **^,a^	1.80 ± 0.05 **^,b^	1.64 ± 0.07 *^,a,b^		≤0.001
*p*-value ^†^		n. a.	0.019	0.123	≤0.001	n. a.	
Apoptosis	0.5 cm^2^/mL	3.90 ± 3.18	9.90 ± 3.63 *^,^**	12.70 ± 3.50 *^,^**	15.20 ± 3.94 *,**	28.10 ± 3.05	≤0.001
	3.0 cm^2^/mL		21.90 ± 3.00 *^,^**	17.40 ± 4.48 *^,^**^,a^	23.70 ± 4.27 *^,a^		≤0.001
*p*-value ^†^		n.a.	≤0.001	0.019	≤0.001	n. a.	
Necrosis	0.5 cm^2^/mL	1.20 ± 0.79	1.00 ± 0.82 **	1.10 ± 1.10 **	2.00 ± 1.33 **	9.60 ± 3.25	≤0.001
	3.0 cm^2^/mL		3.20 ± 2.66 **	3.00 ± 1.49 **	3.50 ± 2.37 *^,^**		≤0.001
*p*-value ^†^		n. a.	0.019	0.009	0.143	n. a.	
Cells with undamaged chromatin	0.5 cm^2^/mL	1968.3 ± 5.42	1956.40 ± 10.66 **^,a^	1963.40 ± 5.60 **^,b^	1931.20 ± 7.44 **,^a,b^	1841.30 ± 11.12	≤0.001
	3.0 cm^2^/mL		1925.70 ± 7.94 *^,^**	1940.40 ± 10.12 **^,a^	1904.80 ± 9.30 *^,^**^,a^		≤0.001
*p*-value ^†^		n. a.	≤0.001	≤0.001	≤0.001	n. a.	

The data are presented as the mean and standard deviation. ^†^ Mann–Whitney test; ^††^ Kruskal-Wallis test; *p* < 0.05. In the rows, the same lowercase letter in superscript indicates a difference between the materials. Symbols: *, statistically significant compared to the negative control; **, statistically significant compared to the positive control; n. a., not acceptable.

**Table 5:** Micronucleus cytome assay results for V79 cell line after exposure to different materials at varying concentrations.

Cytogenetic Damage	Concentration	Negative Control	MTA Fillapex	Bioroot RCS	AH Plus	Positive Control	*p*-Value ^††^
Micronucleus	0.5 cm^2^/mL	2.36 ± 1.29	3.40 ± 1.43 **^,a^	5.10 ± 2.38 **^,b^	6.20 ± 2.04 *^,^**^, a,b^	34.40 ± 3.73	≤0.001
	3.0 cm^2^/mL		4.80 ± 2.66 **^,a^	5.90 ± 1.66 **^,b^	11.50 ± 1.96 *^,^**^,a,b^		≤0.001
*p*-value ^†^		n. a.	0.353	0.436	≤0.001	n. a.	
Nuclear buds	0.5 cm^2^/mL	2.82 ± 1.33	14.90 ± 4.09 *^,^**^,a^	9.60 ± 2.88 *^,^**^,a,b^	16.50 ± 3.10 *^,^**^,b^	27.50 ± 3.85	≤0.001
	3.0 cm^2^/mL		24.30 ± 4.47 *^,a^	15.40 ± 3.24 *^,^**^,a,b^	20.86 ± 1.05 *^,b^		≤0.001
*p*-value ^†^		n. a.	≤0.001	0.002	≤0.001	n. a.	
Nucleoplasmic bridges	0.5 cm^2^/mL	0.91 ± 0.72	0.90 ± 0.88 **	0.80 ± 0.67 **	0.90 ± 0.57 **	6.00 ± 2.00	≤0.001
	3.0 cm^2^/mL		2.10 ± 1.29 **	2.20 ± 1.42 **	2.70 ± 1.48 **		≤0.001
*p*-value ^†^		n. a.	0.043	0.002	0.043	n. a.	
CBPI	0.5 cm^2^/mL	1.81 ± 0.05	1.84 ± 0.06 **	1.83 ± 0.0 **	1.84 ± 0.04 **	1.63 ± 0.03	≤0.001
	3.0 cm^2^/mL		1.69 ± 0.04 **^,a^	1.80 ± 0.05 **^,a,b^	1.77 ± 0.08 ^b^		≤0.001
*p*-value ^†^		n. a.	≤0.001	0.143	0.023	n. a.	
Apoptosis	0.5 cm^2^/mL	3.90 ± 3.18	9.90 ± 3.63 *^,^**	12.70 ± 3.50 *^,^**	15.20 ± 3.94 *^,^**	28.10 ± 3.05	≤0.001
	3.0 cm^2^/mL		21.90 ± 3.00 *^,^**	17.40 ± 4.48 *^,^**^,a^	23.70 ± 4.27 *^,^**^,a^		≤0.001
*p*-value ^†^		n. a.	≤0.001	0.019	≤0.001	n.a.	
Necrosis	0.5 cm^2^/mL	1.20 ± 0.79	1.00 ± 0.82 **	1.10 ± 1.10 **	2.00 ± 1.33 *^,^**	9.60 ± 3.25	≤0.001
	3.0 cm^2^/mL		3.20 ± 2.66 **	3.00 ± 1.49 **	3.50 ± 2.37 *^,^**		≤0.001
*p*-value ^†^		n. a.	0.019	0.009	0.143	n. a.	
Cells with undamaged chromatin	0.5 cm^2^/mL	1968.3 ± 5.42	1956.40 ± 10.66 *^,^**^,a^	1963.40 ± 5.60 *^,^**^,b^	1931.20 ± 7.44 *^,^**^,a,b^	1841.30 ± 11.12	≤0.001
	3.0 cm^2^/mL		1925.70 ± 7.94 *^,^**	1940.40 ± 10.12 **^,b^	1904.80 ± 9.30 *^,^**^,b^		≤0.001
*p*-value ^†^		n. a.	≤0.001	≤0.001	≤0.001	n. a.	

The data are presented as the mean and standard deviation. ^†^ Mann–Whitney test; ^††^ Kruskal-Wallis test; *p* < 0.05. In the rows, the same lowercase letter in superscript indicates a difference between the materials. Symbols: *, statistically significant compared to the negative control; **, statistically significant compared to the positive control; n. a., not acceptable.

## Data Availability

The original contributions presented in the study are included in the article, further inquiries can be directed to the corresponding author.
